# The clustered protocadherin endolysosomal trafficking motif mediates cytoplasmic association

**DOI:** 10.1186/s12860-015-0074-4

**Published:** 2015-11-25

**Authors:** Adam Shonubi, Chantelle Roman, Greg R. Phillips

**Affiliations:** Department of Biology, College of Staten Island, City University of New York, 2800 Victory Blvd, Staten Island, NY 10314 USA; Center for Developmental Neuroscience, College of Staten Island, City University of New York, 2800 Victory Blvd, Staten Island, NY 10314 USA; CUNY Graduate Center, College of Staten Island, City University of New York, 2800 Victory Blvd, Staten Island, NY 10314 USA

**Keywords:** Protocadherins, Adhesion, Cadherin, Endosome, Trafficking, Electron microscopy

## Abstract

**Background:**

Clustered protocadherins (Pcdhs) are a large family of neural cadherin-like proteins encoded by individual exons located within three gene clusters. Each exon codes an extracellular, transmembrane, and proximal cytoplasmic domain. These “variable” regions may be spliced to a constant cytoplasmic moiety encoded at the end of a cluster. Pcdh extracellular domains mediate homophilic cell-cell binding but their cytoplasmic domains cause intracellular retention and may negatively regulate Pcdh cell-cell binding. Pcdhs can be found at the cell surface in neurons and other cells but are also, unlike classical cadherins, prominently trafficked to the endolysosome system. It was previously found that a segment within the variable portion of the Pcdh-γA3 cytoplasmic domain (VCD) was shown to be necessary for endolysosomal trafficking.

**Results:**

Here it is shown that this same VCD segment can mediate cytoplasmic association among Pcdhs from the different clusters. Internal deletions within this VCD region (termed here the VCD motif) that disrupt the association altered trafficking of Pcdh-γA3 in the endolysosomal system while deletions outside VCD motif did not affect trafficking.

**Conclusions:**

The results show that Pcdhs associate cytoplasmically via a motif within the VCD and that this is critical for Pcdh trafficking. Given that truncation at the VCD motif alters endolysosomal trafficking of Pcdhs, the VCD interaction described here may provide new insights into the dynamic nature of Pcdh mediated cell-cell interactions.

## Background

Clustered protocadherins (Pcdh) are a large family of vertebrate neural recognition proteins encoded by three tandem gene clusters (α, β, and γ) [1–3]. Individual exons in the clusters encode different, but related, extracellular, transmembrane and proximal cytoplasmic domains. These can be spliced to constant domain exons at the end of the α and γ clusters [4]. The clustered arrangement allows epigenetic regulation of Pcdh expression, resulting in unique repertoires expressed by different neurons [5–8]. This is thought to be a basis for a recognition “code” [9, 10] in the nervous system that may confer synaptic specificity as well as the specificity of glial [11] and axonal interactions [12].

Recently, it was shown that Pcdhs, in particular the γ subfamily, are involved in same-cell avoidance of dendrites in amacrine cells of the retina and Purkinje cells of the cerebellum [13, 14]. Genetic disruption of the γ cluster in these regions resulted in more dendrite crossings in vivo and in cultures. In other neurons such as cortical pyramidal cells, disruption of the γ cluster caused dendrite defects [15, 16]. Pcdh-γs are also expressed at synapses [17] but with a largely intracellular distribution in synaptic organelles [18, 19].

Pcdh cell-cell binding activity is enhanced by deletion of the cytoplasmic domain [9, 20]. The variable cytoplasmic domain (VCD) for one Pcdh-γ (Pcdh-γA3) was found to contain a ~26 amino acid sequence (termed here the VCD motif) that directs the molecule to the endolysosome system in cell transfection experiments [21, 22]. This motif is almost identical among all γAs and also shows conservation in members of the other Pcdh clusters. It is likely that the mechanism by which Pcdhs engage membranes at sites of cell-cell contact involves endocytosis and endolysosomal trafficking [23, 24].

We report here that the VCD motif in γA3 mediates a novel cytoplasmic cis-association among representatives from all three Pcdh subclusters. Deletions within the γA3 VCD motif abolished interaction and disrupted trafficking to the endolysosome system. The VCD motif has conserved features throughout the three Pcdh gene clusters. Altogether, these results show that the VCD motif in clustered Pcdhs is a cytoplasmic effector that controls Pcdh function.

## Results

### Cytoplasmic association among Pcdh VCDs

**Fig. 1:**
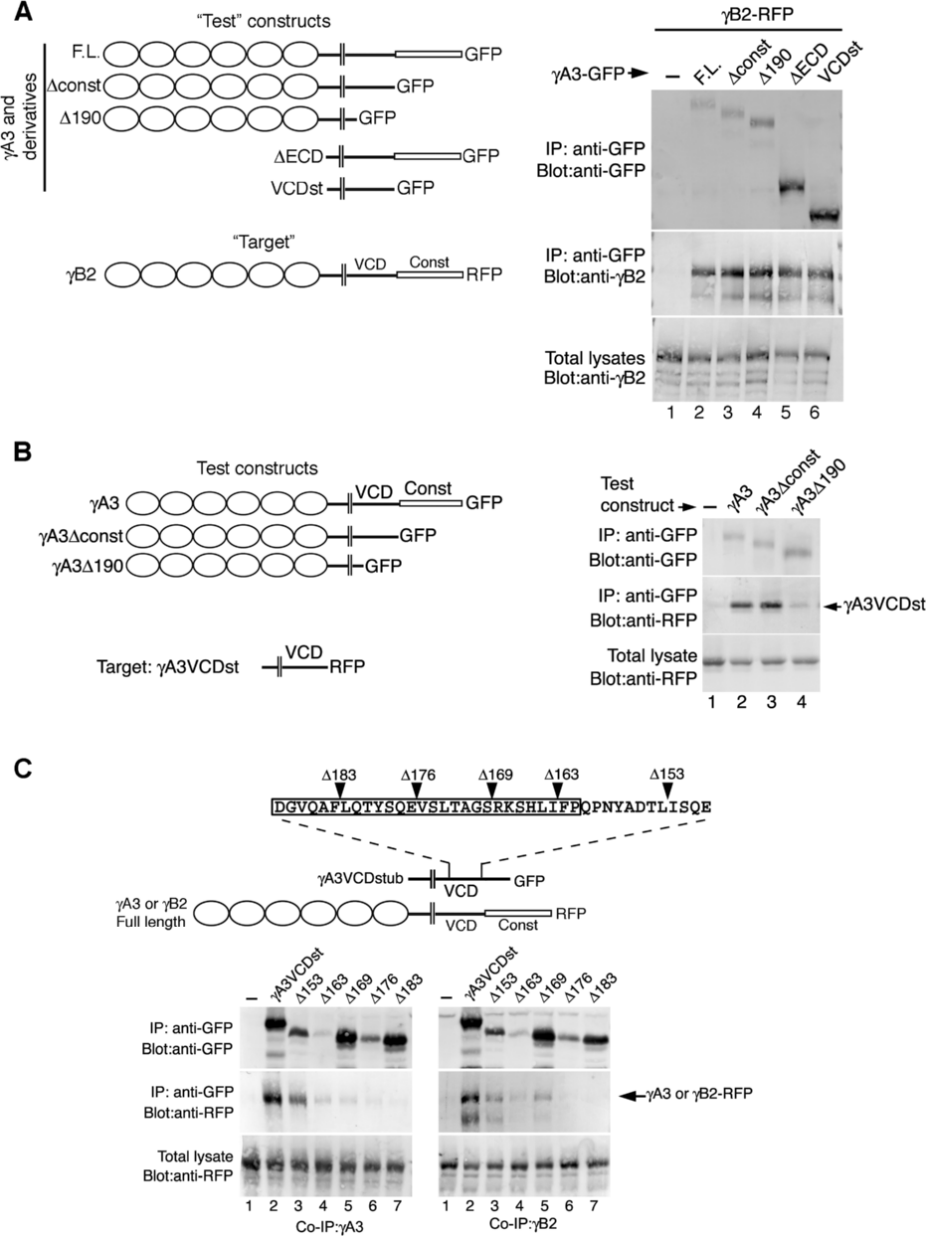
Novel intracellular association of Pcdhs. **a** Indicated “test” constructs derived from γA3 were cotransfected with the γB2 full-length “target”, complexes were immunoprecipitated (IP) with anti-GFP beads, and blots were probed with the indicated antibodies. In the absence of γA3-GFP, γB2-RFP was not precipitated with anti-GFP beads, even though γB2-RFP was present in the total lysate (lane 1). γA3 full-length, constant domain deleted (Δconst), and the larger cytoplasmic deletion (Δ190) of γA3 all coprecipitated with full-length γB2 (lanes 2–4) confirming an extracellular interaction described previously [9]. However, the γA3 extracellular deletion (ΔECD, lane 5) as well as a “stub” construct consisting mostly of the variable cytoplasmic domain (VCDst, lane 6) also coprecipitated with γB2 indicating a novel cytoplasmic association. **b** Indicated γA3 test constructs derived were coprecipitated with the γA3 VCD stub. The stub associated with full length and constant domain deleted γA3 (lanes 2 and 3) but not with γA3Δ190 (lane 4). **c** The γA3 VCD stub was truncated at the indicated points (arrowheads) and tested for coprecipitation with full-length γA3 or γB2-RFP. Boxed region indicates the previously mapped trafficking motif (VCD motif). Truncation into the VCD motif eliminated coprecipitation with full length γA3 and γB2

Pcdhs are known to form a complex in *cis* that involves extracellular interaction [9, 10, 25, 26]. This extracellular cis interaction can influence Pcdh surface delivery [10, 25, 26]. An intracellular interaction among Pcdhs was also identified but not localized to a particular domain or set of residues [25]. We sought to determine the nature of the cytoplasmic interaction that might also participate in Pcdh complex formation. Full length γA3-GFP and truncated variants were cotransfected with full length γB2-RFP (Fig. 1a), complexes were immunoprecipitated with anti-GFP beads, and analyzed by immunoblotting. In the absence of a GFP construct, γB2-RFP was not precipitated with anti-GFP beads (Fig. 1a, lane 1). Full length γA3-GFP coprecipitated γB2-RFP as did constant domain deleted γA3-GFP (Δconst), as well as a deletion of most of the cytoplasmic domain (Δ190) confirming the extracellular interaction described previously [9]. However, we found that an extracellular deletion of γA3-GFP (ΔECD) also coprecipitated with γB2-RFP (Fig. 1a, lane 5) indicating an intracellular association between these two Pcdhs. This association was equally effective with a γA3 “VCD stub” construct which lacked the constant domain in addition to the extracellular domain (VCDst; Fig 1a, lane 6). To assess whether the γA3 VCD can also complex with itself, GFP fused full length, constant domain deleted (Δconst) or constant domain plus VCD deleted (Δ190) γA3 was cotransfected with the γA3 VCD stub fused to RFP. Full length γA3 and γA3Δconst coprecipitated with the VCD stub while γA3Δ190 did not (Fig. 1b).

Endogenous Pcdh-γs are largely intracellular in synaptic compartments [18, 19]. Consistent with this, γA3-GFP is trafficked to organelles in primary neurons and to late endosomes in HEK293 cells [20, 21]. Previously, carboxy-terminal deletions mapped this trafficking activity to the VCD motif [22]. Here, similar carboxy-terminal deletions of the γA3 VCD stub, fused to GFP, were prepared and assayed for their ability to co-immunoprecipitate with full length γA3-RFP or γB2-RFP (Fig. 1c). Deletions of the VCD stub just prior to the VCD motif (Δ153) still allowed coprecipitation with full length γA3 (Fig. 1c, left, lanes 2–3) or γB2 (Fig. 1c, right, lanes 2–3). Deletions closer to, or within the VCD motif, (Δ163-Δ183) greatly reduced or eliminated binding to both full length molecules (Fig. 1c, lanes 4–7).

**Fig. 2:**
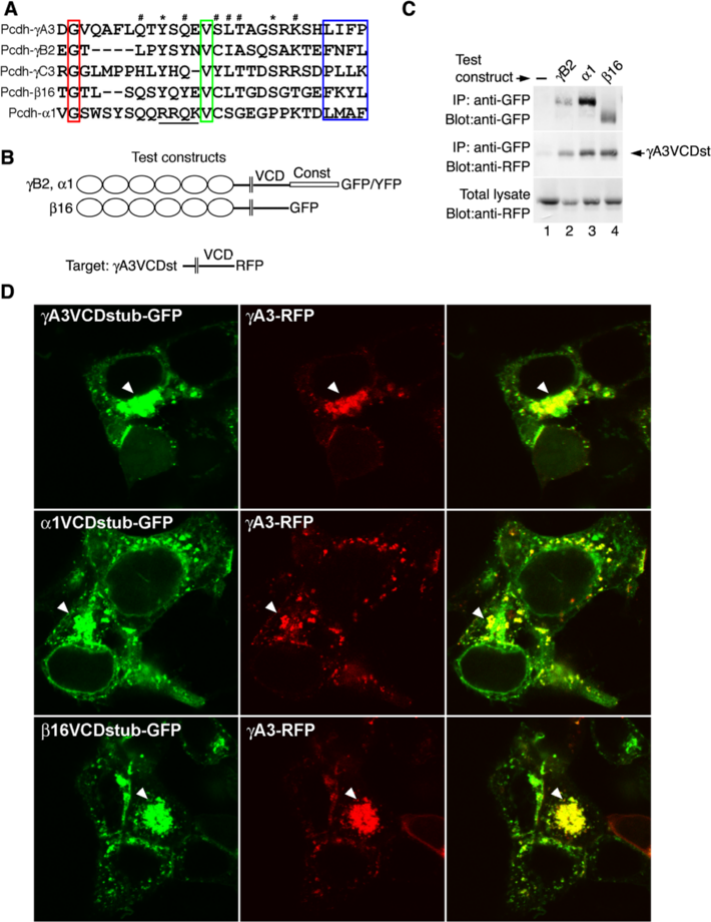
γA3 VCD stub associates with Pcdhs from other clusters. **a** Manual alignment of the VCD motifs from the indicated Pcdhs. Boxes indicate residues used to anchor alignment. The unique basic sequence in α1 is underlined. Asterisks and pound signs indicate residues conserved in 4 out of 5 and 3 out of 5 sequences, respectively. **b** Constructs used for coimmunoprecipitation. **c** The γA3 stub is coprecipitated with full length Pcdhs from other clusters. **d** VCD stubs (green) from γA3 (top), α1 (middle) and β16 (bottom) colocalize (arrowheads) full length γA3 in transfected cells

The γA3 VCD motif is nearly identical for all γAs and shares some homology with γB2 while the γBs are much more variable in this region amongst themselves [22]. Manual inspection of amino acid sequences from representatives of the other mouse Pcdh subclusters reveals sequences resembling the VCD motif in other clustered Pcdhs. Alignment of a conserved glycine residue (residue 740 for γA3, 744 for γB2, 749 for γC3, 743 for α1, and 744 for β16) reveals additionally conserved residues throughout the VCD motif (Fig. 2a). Viewed in this way, a number of features can be observed. After the conserved glycine there is a region with conserved glutamine, tyrosine and serine residues. Of note, the α1 sequence contains a basic segment (Fig. 2a, underlined) that the other Pcdhs lack. A strongly conserved valine residue (green box, Fig. 2a) is followed by a region with basic residues, serines and threonines. Finally at the end of the motif is a region containing hydrophobic and aromatic amino acids (blue box, Fig. 2a). VCD motifs were not detected in Pcdh-αC1, −αC2, −γC4 or -γC5.

Because γA3 cytoplasmic association with itself and γB2 depended on an intact VCD motif, we sought to determine if the γA3 VCD might interact with other clustered Pcdhs. The γA3 VCD stub, fused to RFP was cotransfected with full length γB2, α1 or β16, complexes were isolated with anti-GFP beads and probed with anti-GFP or anti-RFP. The γA3 VCD stub was found to readily coprecipitate with γB2, α1 and β16 (Fig 2c). Full length γA3-RFP was also found to colocalize with the VCD stubs from γA3, α1 and β16 in transfected cells (Fig. 2d, arrowheads) indicating that VCD-VCD association is likely to be an important aspect for the function of all clustered Pcdhs.

### VCD motif deletions disrupt Pcdh-γ endolysosomal trafficking

**Fig. 3:**
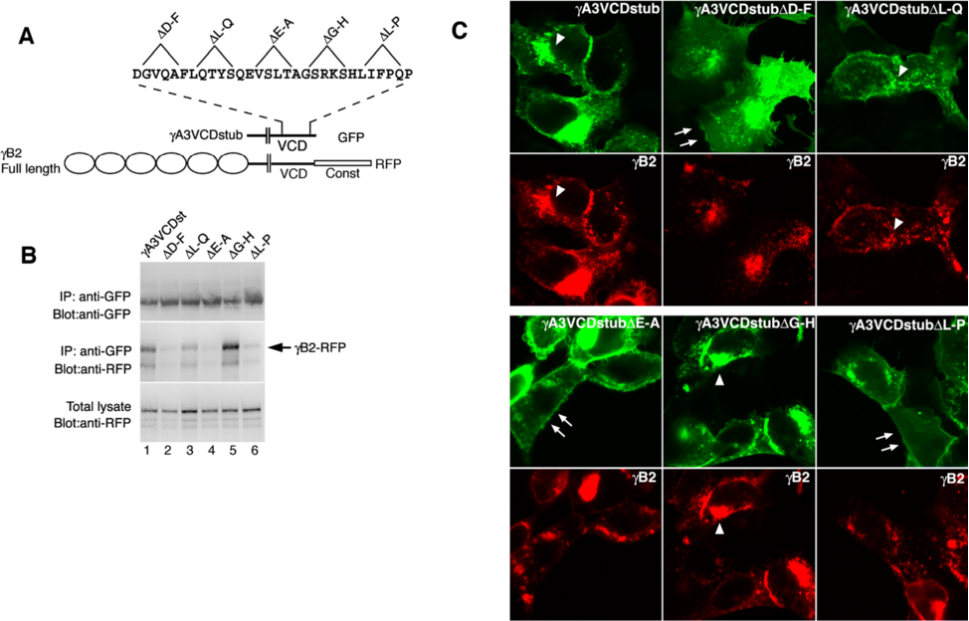
Internal deletions within the γA3 VCD motif affect association with full length γA3. **a** Six amino acid deletions in the γA3 VCD motif within the context of the VCD stub. **b** Coprecipitation of the VCD stub deletions with full length γB2. Deletions ΔD-F, ΔE-A, and ΔL-P significantly reduced association while other deletions had little or no effect. **c** Colocalization of VCD stub deletions with γB2. The γA3 VCD stub colocalized with full length γB2 in cells (arrowheads). Mutations that reduced VCD stub coprecipitation exhibited less colocalization with full length γB2 and could be found diffusely at the cell surface (double arrows)

To further confirm that the VCD motif mediates cytoplasmic Pcdh association, internal deletions of 6 amino acids were constructed spanning the entire motif shown previously to be active for trafficking (Fig. 3a). Three out of the 5 internal deletions (ΔD-F, ΔE-A, ΔL-P; Fig. 3b, lanes 2, 4, 6) exhibited markedly reduced coprecipitation with γB2 indicating that the active domain spans the VCD motif. In contrast, other deletions (ΔL-Q and ΔG-H; Fig. 3b, lanes 3 and 5) had less of an effect on coprecipitation with γB2. Thus the VCD motif spans a site that mediates VCD interactions.

These co-immunoprecipitation results were reflected in the ability of the γA3 VCD stub mutants to colocalize with γB2-RFP in cotransfected cells. The wild-type γA3 VCD stub colocalized with γB2-RFP (Fig. 3c, arrowheads) as did the ΔG-H mutant VCD stub (Fig. 3c). The ΔL-Q deletion, which exhibits weaker association by co-immunoprecipitation, exhibited some colocalization of γB2. In contrast, most of the mutants that lacked significant VCD binding activity (ΔD-F, ΔE-A, ΔL-P) exhibited diffuse cell surface distribution (double arrows, Fig. 3c) with less colocalization with γB2-RFP.

**Fig. 4:**
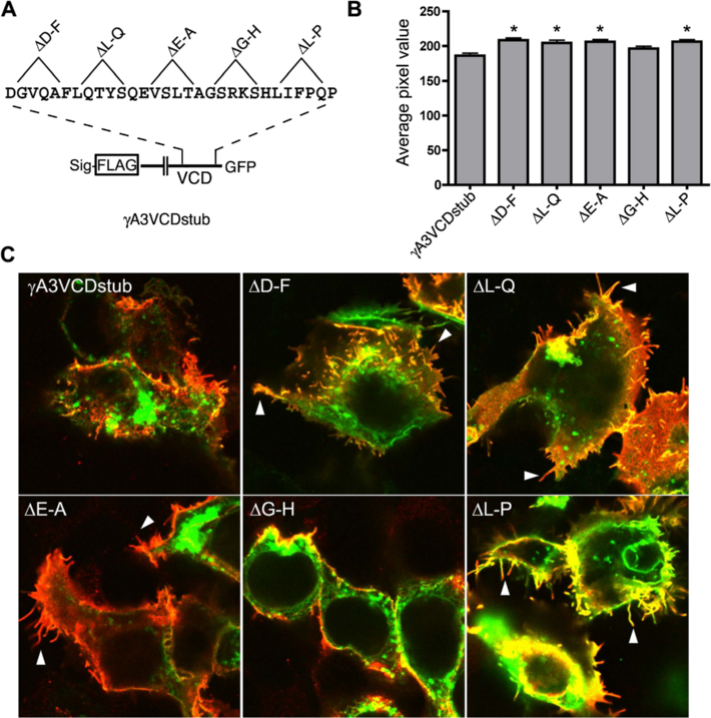
Internal deletions within the VCD motif affect surface trafficking. **a** Internal deletions within the VCD stub and location of the FLAG epitope used to detect surface delivery. **b** Quantification of surface delivery. Average pixel value of surface FLAG immunoreactivity for each construct was calculated from 50 cells per condition ± SEM. Significance determined by *t*-test. * indicates *p* < 0.001. **c** Surface labeling (red) of VCD stub GFP (green) and indicated deletions. Constructs more highly expressed on the surface resulted in more prominent filopodia (arrowheads) labeled with the FLAG antibody

To more precisely study the surface delivery of γA3 VCD stub mutants, we performed surface labeling experiments using the extracellular FLAG epitope present on the stub constructs (Fig. 4a). Quantitative analysis of surface FLAG staining showed that mutations that reduced VCD association (ΔD-F, ΔL-Q, ΔE-A, ΔL-P) caused an increase in surface delivery. The ΔG-H mutation did not exhibit reduced VCD association as compared to wild type (see Fig. 3) and accordingly, had surface levels similar to the wild type γA3 VCD stub. Increased surface expression of the mutant VCD stubs corresponded to more prominent filopodia (arrowheads in Fig. 4c).

**Fig. 5:**
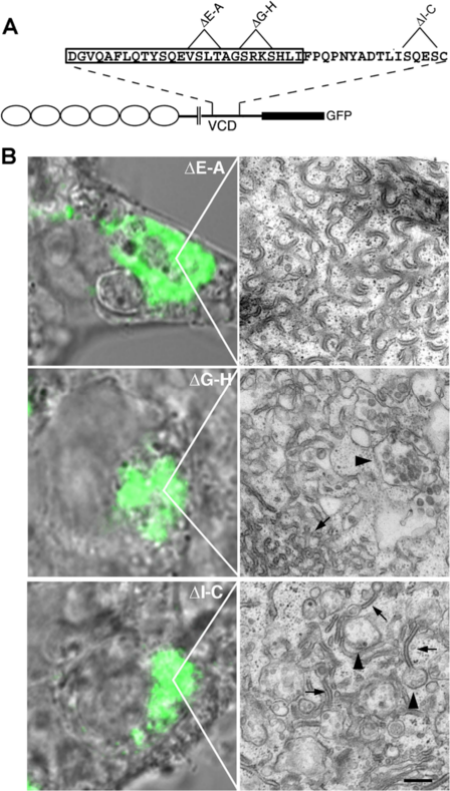
**a** VCD motif deletions affect full length γA3 trafficking. Mutations that reduced (ΔE-A) or did not affect (ΔG-H) VCD association were generated within the context of full length γA3-GFP and evaluated for intracellular trafficking by correlative light and electron microscopy. Another mutation outside the VCD motif (ΔI-C) was also tested. **b** ΔE-A caused the molecule to accumulate in wavy structures with no multivesicular bodies observed. ΔG-H was associated with wavy membranes (arrow) as well as distorted enlarged multivesicular bodies (arrowhead). In contrast, a deletion outside the VCD motif (ΔI-C) allowed correct trafficking to multivesicular bodies (arrowhead) and tubules (arrow) previously observed for intact γA3. Bar =200 nm

**Fig. 6:**
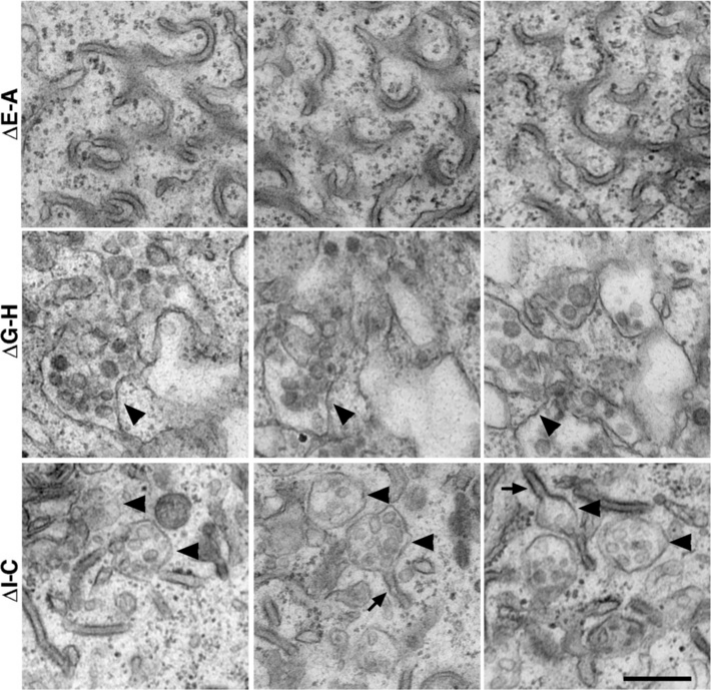
Serial sections through organelles accumulations induced by the ΔE-A, ΔG-H or ΔI-C full length constructs. ΔE-A accumulated in reticular like structures with no vesicular profiles evident. In contrast ΔG-H exhibited some vesicular profiles that were larger and more amorphous than those produced by ΔI-C, which had multivesicular bodies (arrowheads) and associated tubules (arrows) very similar to those found in wild type Pcdh-γA3 transfected cells [21, 22]. Bar = 250 nm

We asked how the deletion mutations that disrupt VCD association can affect endolysosomal trafficking of full length γA3 (Fig. 5). Correlative light and electron microscopy of transfected wild-type γA3 previously revealed the accumulation of ~250 nm multivesicular bodies and associated tubules [21, 22] while untransfected cells lacked these organelles. In contrast, in cells accumulating full length γA3-GFP containing the ΔE-A mutation, there were never multivesicular bodies or tubules associated with the area of GFP expression. Rather, wavy structures reminiscent of an expansion of ER (Fig. 5b, top) were observed. In cells expressing full length γA3 containing the ΔG-H mutation, which did not affect VCD-VCD interaction, there were large misshapen multivesicular structures of approximately ~500 nm with few associated tubules (arrowhead, Fig. 5b, middle), as well as wavy expanded ER-like membranes (arrow). In contrast to the trafficking defects observed with deletions made within the VCD motif, when a deletion was made outside this motif (ΔI-C; Fig. 5b, bottom), normal trafficking of the molecule to ~250 nm multivesicular bodies (arrowheads) and tubules (arrows) was observed as shown previously [21, 22]. Serial sectioning of each sample confirmed the abnormal trafficking of the ΔE-A and ΔG-H mutations (Fig. 6). γA3 ΔE-A showed a reticular accumulation with no vesicular structures observed, while γA3 ΔG-H exhibited vesicles that were abnormally formed and that generally lack the tubules found in wild type (not shown) and the γA3 ΔI-C mutation. In no instances were any abnormal vesicles or organelle accumulations observed in non-transfected cells. The combined results suggest that intracellular association via the VCD motif likely plays a role in trafficking, and hence function, of γA3 and likely other Pcdhs. Lack of VCD association could result in mistrafficking and accumulation in abnormal organelles.

## Discussion

Pcdhs can promote homophilic cell-cell interaction in in vitro assays [9, 10], consistent with their resemblance to classical cadherins. However, their cell surface expression is negatively regulated by their cytoplasmic domains [9, 20], making their adhesion in vitro somewhat weaker than classical cadherins [27]. Endogenous Pcdhs are also located mostly within intracellular compartments [19], mirroring the intracellular localization of expressed Pcdhs. Because of their intracellular retention, it has been difficult to reconcile an adhesive role for the Pcdhs as stabilizers of cell-cell junctions. Neuron-glia interactions can be stabilized by Pcdhs [11] but other studies showed a role in dendrite self-avoidance [13]. These different results highlight the importance of cell biological studies that address the unique and dynamic mode of Pcdh mediated cell-cell interactions at the molecular level.

Cytoplasmic interactions are likely be the key to understanding different modes of Pcdh cell-cell binding. Information on Pcdh cytoplasmic interactions is still limited when compared to classical cadherins. The Pcdh-γ constant cytoplasmic domain was previously shown to interact with PDCD10 (programmed cell death 10) with a role in neuron survival [28]. More recently, the Pcdh-γ constant domain was shown to bind focal adhesion kinase (FAK) [15] and phospholipids [16] and that these interactions can be modulated by protein kinase C (PKC) with dramatic consequences for dendrite arborization. How the VCD interactions described in the present study might affect these constant domain functions remains to be determined but the combined data point to an increasingly complex network of cytoplasmic interactions for the clustered protocadherins.

## Conclusions

How can we reconcile Pcdh homophilic binding at the cell surface with their prominent intracellular trafficking in the endolysosome system? Based on our findings here and previous studies [18–22], it is possible that Pcdh engagement at the surface may trigger internalization of the adhesive complex in instances where Pcdhs might be necessary for anti-adhesion. On the other hand, Pcdh pro-adhesion might be activated if their internalization mechanisms were to be blunted. The results from the present study indicate that modulation of interactions among Pcdh VCDs could be involved in determining how Pcdhs operate at cell-cell interfaces.

## Methods

### cDNA constructs

The plasmids encoding Pcdh-γA3-GFP, extracellular deleted γA3-GFP (ΔECD), constant domain deleted γA3-GFP (Δconst), γA3 with the constant and most of the variable cytoplasmic domain removed (Δ190), and the γA3 VCD stub, have been described [18, 20, 22, 29]. Pcdhs γB2-RFP and γA3-RFP were provided by Dr. Joshua Weiner, α1-GFP by Dr. Qiang Wu, and β16-GFP by Dr. Dirk Junghans. The plasmids encoding the transmembrane and VCD stubs of α1 and β16 were generated by amplification of nucleotides corresponding to amino acids 684 to 796 of mouse α1 and 665 to 802 of mouse β16 coding sequences. The segments were subcloned in frame into the BamH1-Age1 sites of the plasmid originally used to construct extracellular deleted Pcdh-γA3 [29]. The resultant stub constructs have the signal sequence from CD97a, followed by a FLAG tag, followed by the transmembrane domain, VCD and GFP. The completed VCD stub constructs were also subcloned into pDsRed2-n1.

### Cell transfection

HEK293 cells (ATCC CRL-1573) were grown in DMEM containing 10 % FBS. Cells were transfected by calcium phosphate precipitation. Cells were grown overnight prior to assaying.

### Immunoprecipitation

Transfected cells were lysed with 1 % Triton X-100 in 20 mM Tris (pH 7.4), 150 mM NaCl and lysates cleared by centrifugation. Pcdh complexes were immunoprecipitated with anti-GFP coupled agarose beads (MBL), electrophoresed and transferred. Blots were probed with anti-GFP, anti-dsRed (Clontech), or anti-Pcdh-γB2 (Neuromab).

### Surface labeling

Cells transfected with VCD stub constructs were fixed and labeled with anti-FLAG (clone M2, Sigma) at 1:500 dilution in phosphate buffered saline (PBS) containing 3 % bovine serum albumin (Fraction V, Sigma) in the absence of permeabilizing detergent. Cells were washed and stained with goat anti-rabbit IgG Alexa 548 (ThermoFisher) conjugated secondary antibodies in the same buffer, washed and mounted. Surface labeling was visualized using the Leica SP2 confocal microscope with pinhole settings at ~ 231 μm to collect light from the entire cell surface. All VCD stub mutant images were acquired using the same laser power, gain and offset. Surface labeling was quantified in ImageJ by measuring the mean gray value for 50 cells each condition. Values were then averaged and significance determined by *t*-test (*p* < 0.001).

### Imaging

For confocal imaging, transfected cells were fixed in 4 % paraformaldehyde/4 % sucrose in phosphate buffered saline, washed and mounted. Confocal imaging was performed on a Leica SP2 confocal microscope (Advanced Imaging Facility, College of Staten Island). Correlative light and electron microscopy was performed as described [30].

## Abbreviations

DMEM: Dulbecco’s modified eagle medium
ECD: extracellular domain
ER: endoplasmic reticulum
FAK: focal adhesion kinase
GFP: green fluorescent protein
HEK293: human embryonic kidney 293 cells
Pcdh: clustered protocadherin
PKC: protein kinase C
RFP: red fluorescent protein
VCD: variable cytoplasmic domain
